# Recent Applications of Polymer Supported Organometallic Catalysts in Organic Synthesis

**DOI:** 10.3390/molecules15096306

**Published:** 2010-09-07

**Authors:** Nina Kann

**Affiliations:** Organic Chemistry, Department of Chemical and Biological Engineering, Chalmers University of Technology, SE-41296 Göteborg, Sweden; E-Mail: kann@chalmers.se; Tel.: +46-31-772-3070; Fax: +46-31-772-3858

**Keywords:** polymer supported, organometallic, solid phase synthesis, cross-coupling, metathesis, allylic substitution, transfer hydrogenation, aryl amination

## Abstract

Recent developments concerning the application of polymer supported organometallic reagents in solid phase synthesis are reviewed, with a special focus on methodology for carbon-carbon formation. Examples of reactions that are covered include the classical Suzuki, Sonogashira and Heck coupings, but also aryl amination, epoxide opening, rearrangements, metathesis and cyclopropanation. Applications in the field of asymmetric synthesis are also discussed.

## Abbreviations

acac: acetylacetonato
AIBN: azoisobutyronitrile
BSA: *N*,*O*-bis(tri-methyl silyl)-acetamide
DMA: *N*,*N*-dimethylacetamide
DPEN: 1,2-diphenyl-ethylenediamine
DVB: divinylbenzene
NHC: *N*-heterocyclic carbene
PEG: polyethylene glycol
PHOX: diphenylphosphinooxazoline
PS: polystyrene
TBAA: tetrabutyl-ammonium acetate
TEMPO: 2,2,6,6-tetramethyl-piperidin-1-oxyl
TOF: turnover frequency
TON: turnover number

## 1. Introduction

This review covers literature concerning the application of polymer bound organometallic reagents in organic synthesis from 2007 until May 2010; for earlier reports in this area we refer to comprehensive reviews covering the time up to 2007 [1,2,3,4,5,6,7,8]. Likewise, solid phase organometallic chemistry where the substrate, rather than the catalyst, is attached to the support is not included, but reviews summarizing this field are available [9,10,11]. Due to the plethora of literature on the topic of solid supported metal complexes, in particular palladium-containing catalysts, we have chosen to make some restrictions in the material covered. Thus, catalysts for oxidation and reduction are not discussed here, with the exception of a few selected asymmetric processes such as hydrogenation. We instead refer to a recent review by Beligny and Rademann [12], dealing with both metallic and non-metallic oxidants attached to polymeric supports. Also, we limit this report to the use of non-soluble polymeric supports, thus excluding organometallics attached to silica, soluble polymers and dendrimers, although many interesting new supported catalysts have appeared using such materials also. The review does not aim to be exhaustive, but rather reflects a personal selection of practical and efficient methods with an application towards organic synthesis, and contributing something new in this field.

Much of the recent focus in the area of supported organometallic reagents has been on the ability to recycle the catalyst, important from a green chemistry point of view, and also to limit the extent of leaching of the metal from the solid support. Thus, special care has been taken in including these aspects in the discussion of the individual findings. The review has been divided according to the metal used and further sub-divided according to the application in organic synthesis.

## 2. Cobalt

### 2.1. Ring Opening of Epoxides

**Scheme 1:**
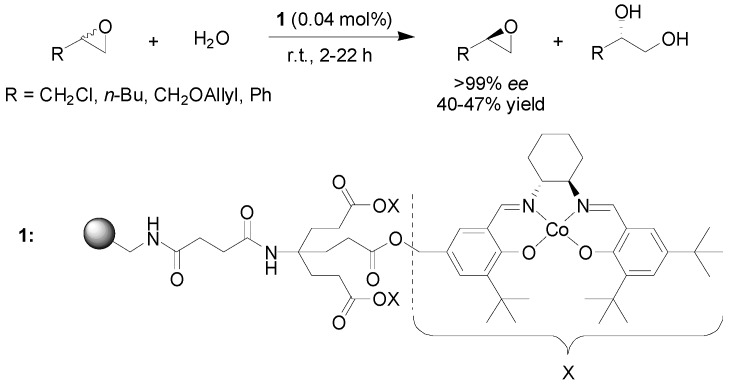
Hydrolytic kinetic resolution of epoxides using a polymer-supported (salen)-cobalt catalyst.

Kinetic resolution of epoxides via hydrolysis has been achieved with a (salen)Co catalyst attached to a dendronized polystyrene support, as reported by Weck and co-workers [13]. The dendron linker allows the attachment of three units of the cobalt-salen catalyst in close proximity, enhancing cooperative interactions and increasing the local catalyst concentration, thus allowing for the use of a significantly lower catalyst loading than earlier achieved using a polymer-supported catalyst for this reaction (Scheme 1). 

Four different epoxide substrates were investigated. Pre-activation of the catalyst [*i.e.* oxidation from Co(II) to Co(III) in the presence of air and acetic acid], followed by the addition of epoxide and water, afforded essentially enantiomerically pure epoxide with yields in the range of 40-47% (maximum theoretical yield 50%) depending on the substitution pattern of the epoxide. Recycling of the supported catalyst was also investigated, showing that although the catalytic activity dropped somewhat after a few cycles, the enantioselectivity of the reaction was not affected much.

## 3. Copper

### 3.1. Cycloaddition Reactions

**Scheme 2:**
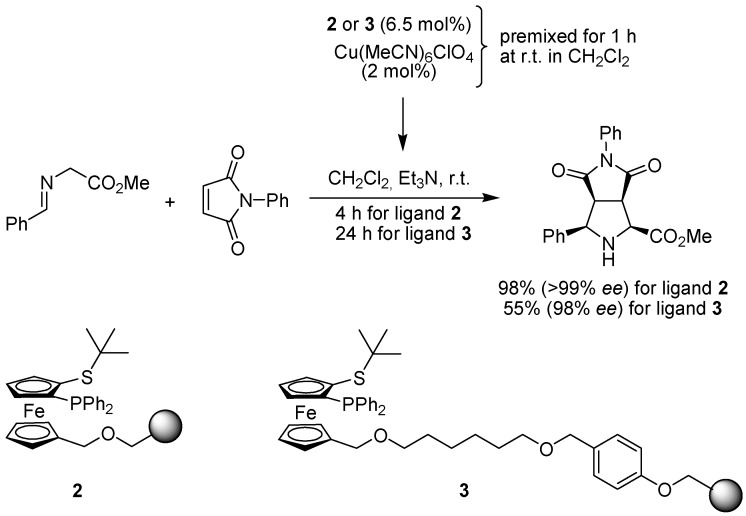
Application of polymer-bound Fesulphos ligands in the copper-catalyzed 1,3-dipolar cycloaddition of an imine to *N*-phenylmaleimide.

Carretero and co-workers have earlier developed a class of ferrocenes, substituted with *tert*-butyl-sulfide and different phosphines, to be used as ligands in metal-catalyzed asymmetric transformations [14]. One of these, the so called Fesulphos ligand, was subsequently equipped with two different handles to enable attachment to Wang resin as well as Merrifield resin and applied in the copper-catalyzed 1,3-dipolar cycloaddition of azomethine ylides (Scheme 2) [15]. Copper coordination to the polymer-bound ligands was found to be slower than the corresponding preparation in solution, requiring one hour rather than a few seconds. However, the polymer-supported catalyst prepared from ligand **2** then performed very well in the cycloaddition reaction of various imines with *N*-phenyl-maleimide and methyl fumarate, giving the cyclized products in high yield and excellent enantioselectivity. Somewhat surprisingly, the use of Fesulphos ligand **3**, attached to a Wang resin via a spacer, afforded the same high stereoselectivity, but markedly lower yields. Ligand **2** was also applied in the palladium-catalyzed asymmetric allylic substitution with good results.

**Scheme 3:**
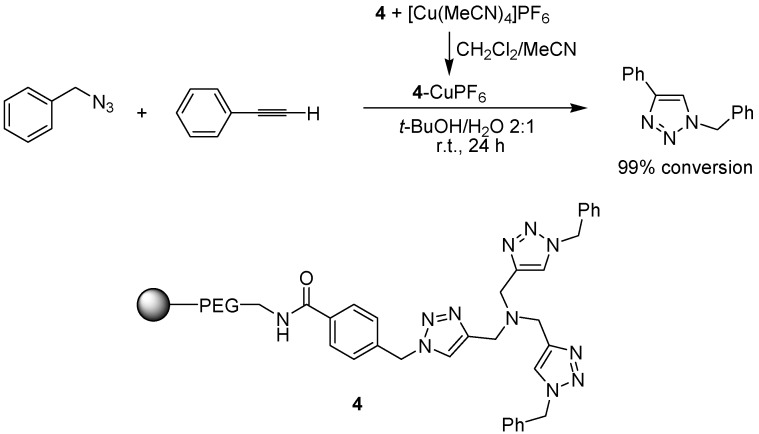
A polymer-supported copper-tris(triazolyl) complex as a catalyst for the cycloaddition of an azide to an alkyne.

The click reaction, *i.e.* the copper-catalyzed azide-alkyne cycloaddition (also called CuAAC), to form triazole products, is an extensively used method for creating a link between two molecules due to the simple reaction conditions and the high yields obtained [16]. Chan and Fokin have developed a Cu(I)-stabilizing tristriazole ligand, prepared via the click reaction, that in turn functions as a catalyst for CuAAC-type reactions [17]. To minimize copper contamination of the product, the ligand was connected to a NovaSyn® TG amino resin (a polystyrene-polyethylene glycol support) and evaluated in the click reaction of phenyl acetylene and benzyl azide (Scheme 3) [18]. Copper(I) was preloaded onto the resin-tethered ligand via washing with a solution of a copper salt. The click reaction itself gave essentially quantitative yield using a wide range of solvents. Leaching of copper was minimal, and the polymer-bound ligand could be recycled up to ten times with only a small decrease in efficiency. 

### 3.2. Other Copper-Catalyzed Reactions

**Scheme 4:**
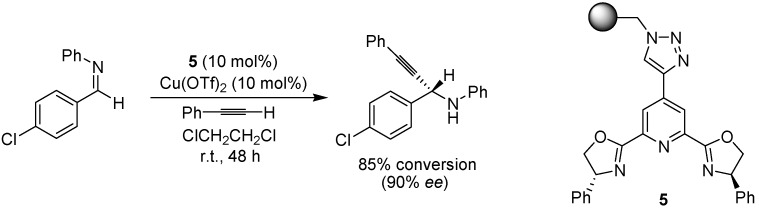
Copper-catalyzed enantioselective alkynylation of an imine using a tethered copper-pybox complex.

Moberg, Levacher and co-workers have developed an efficient protocol for the asymmetric alkynylation of imines [19,20], using a copper-pybox ligand (**5**, Scheme 4), where the ligand was attached to the polystyrene support via a click reaction. Enantioselectivities were somewhat lower than the corresponding reaction in solution, but a range of different imines and alkynes could be coupled with good to excellent conversion.

**Scheme 5:**
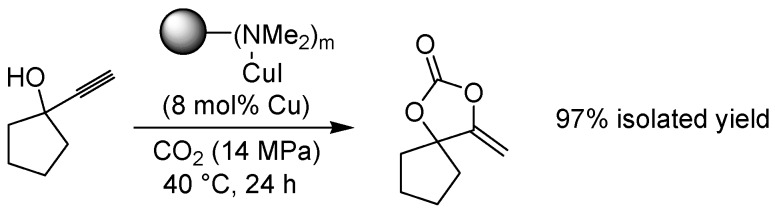
Fixation of carbon dioxide in the form of a cyclic carbonate, catalyzed by a polymer-supported Cu(I)-amine complex.

An interesting derivatization of propargylic alcohols via treatment with supercritical carbon dioxide has been reported on solid support by Jiang *et al*., who applied copper(I) iodide bound to a dimethylamino-polystyrene resin to form α-alkylidene cyclic carbonates (Scheme 5) [21]. Optimum CO_2_-pressure for the reaction was found to lie in the range of 14-18 MPa, producing a wide selection of cyclized products in good yields. The reaction was limited to terminal secondary propargylic alcohols with aliphatic substitutents; no product was obtained when 2-phenyl-3-butyn-2-ol was used as the substrate, nor when primary or internal alkynes were applied in the reaction. A brief mention shall also be made of a report by Cai and co-workers, showing that copper(II) coordinated to a polymer-bound proline can catalyze the cross-coupling of oximes with arylboronic acids in reasonable yields [22].

## 4. Ruthenium

### 4.1. Metathesis

**Scheme 6:**
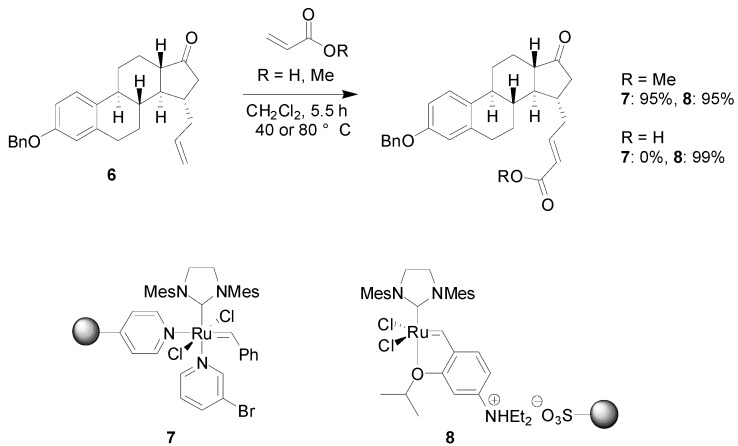
Ruthenium-catalyzed metathesis for the preparation of 17β-hydroxysteroid dehydrogenase type 1 inhibitors.

Kirschning and co-workers have investigated the use of two different polymer bound ruthenium carbenes as metathesis catalysts in the derivatization of a steroid scaffold, with the aim of preparing 17β-hydroxysteroid dehydrogenase type 1 inhibitors [23]. Estrone derivative **6** (Scheme 6), with a pendant allyl group in the C15 position, was subjected to metathesis reactions using a variety of functionalized alkenes, in combination with two different solid-supported Grubbs-type catalysts. In complex **7**, the ruthenium carbene is coordinated to a polymer bound pyridine, while in **8**, the linkage to the polymer is of ionic character. Interestingly, when methyl acrylate was used as the alkene, both reagents performed equally well, while for acrylic acid, only supported catalyst **8** afforded the desired product. In many cases, the reactivity of **8** was found to be similar to the soluble counterpart and ruthenium contamination of the product was much lower than for the homogeneous reaction. Acrylic amides, styrene and vinyl acetate were also applied in the reaction, albeit with varying results.

### 4.2. Asymmetric Transfer Hydrogenation

**Scheme 7:**
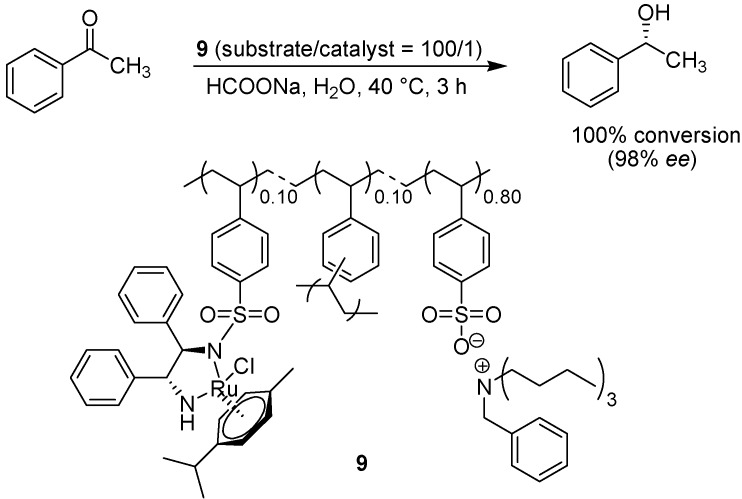
Asymmetric transfer hydrogenation employing chiral polymer supported (1,2-diamine monosulfonamide)-ruthenium complexes.

Haraguchi, Itsuno and co-workers have described a successful method for the asymmetric transfer hydrogenation of imines using a hydrophobic polymer-supported *N*-toluenesulfonyl-1,2-diphenyl-ethylenediamine (TsDPEN) ruthenium catalyst [24]. By incorporating pendant quaternary ammonium sulfate groups onto the polymer backbone, the polymer was rendered hydrophilic and the transfer hydrogenation of ketones such as acetophenone could be carried out in water (Scheme 7) [25]. The degree of cross-linking was also investigated, but found to be of lesser importance as even the use of highly cross-linked polymer (20% DVB) as the catalyst support afforded product in high yield. 

### 4.3. Halogenation

The Kharasch reaction involves the addition of halogenated compounds to an alkene, in general catalyzed by a ruthenium complex in the presence of a radical initiator such as AIBN (azoisobutyronitrile) [26]. Oe and Uozumi have shown that this reaction can be carried out in water, without the addition of AIBN, with the help of a polymer-supported ruthenium catalyst, thus affording an atom-economic and ‘green’ functional group transformation [27]. 

**Scheme 8:**
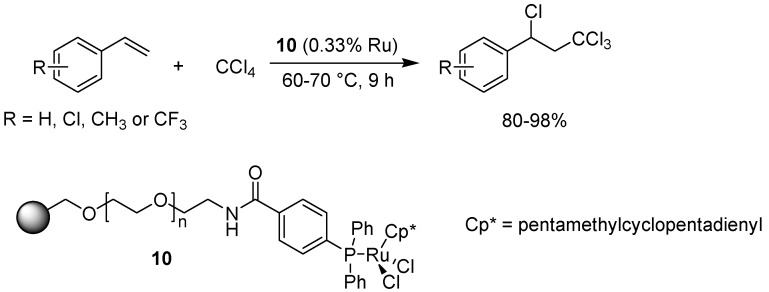
The Kharasch reaction, catalyzed by a polymer-bound ruthenium species in water.

The catalytic system consisted of a ruthenium(III)(pentamethylcyclopentadienyl)dichloride attached to a PS-PEG backbone via a polymer-supported phosphine. A variety of different styrene derivatives were investigated with good results (Scheme 8), affording the addition products in up to 98% yield. Aliphatic alkenes could also be used under these conditions, albeit with a somewhat lower yield. Recycling of the catalyst did not affect the catalytic activity to any extent.

### 4.4. Cyclopropanation

**Scheme 9:**
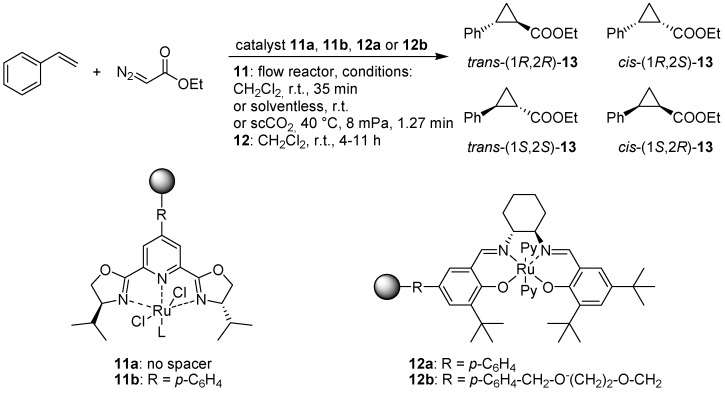
Asymmetric cyclopropanation using polymer bound Ru-pybox (**11**) and Ru-salen (**12**) complexes.

Luis and co-workers have investigated the use of polymer-bound ruthenium-pybox complexes in the cyclopropanation of styrene with diazoacetate (Scheme 9) [28]. 

The catalysts were prepared via polymerization of vinylic monomers of the ligands, with and without an aromatic spacer, inside monolithic minireactors in stainless steel columns, which were subsequently treated with a solution of dichlororuthenium(II) *p*-cymene. In the case of catalyst **11a**, different degrees of cross-linking were also investigated. Minireactors were used to protect the air-sensitive complex from oxygen and to exploit the other advantages of flow reactors such as a large surface area and controlled reaction conditions. Cyclopropanation using **11a** with dichloromethane as the solvent afforded the desired products **13** in up to 50% yield, with a 4:1 *trans:cis* ratio and moderate enantioselectivities, with dimerization of the ethyl diazoacetate being the main side reaction and accounting for the moderate yield. The stereoselectivity was comparable to that obtained for a homogeneously catalyzed reaction using a pybox-Ru complex. The polymer-supported catalyst could be re-used a number of times, without any marked deterioration of the catalytic activity. Solventless conditions afforded up to 72% yield with **11a**, and the use of supercritical carbon dioxide as solvent in conjunction with complex **11b**, also gave good results, showing that the procedure could be carried out in a more environmentally friendly fashion. Likewise, Jones and colleagues have studied the same reaction but using a polymer bound salen-Ru catalyst [29]. Here also two different spacers were used between the complex and the catalyst, a more rigid aromatic linker for **12a** and a flexible ethylene glycyl linker in the case of **12b**. Both catalysts performed better than a silica-supported counterpart, and the yields using the polymer bound catalysts were comparable to the corresponding reactions using a soluble catalyst. The more rigid polymeric catalyst **12a** gave somewhat inferior results in terms of stereoselectivity as compared to **12b**, although the catalytic activity was similar. For catalyst **12b**, diastereoselectivities and enantioselectivities were high (*trans/cis* ratio: 10.9, *trans*: 95% *ee*, *cis*: 92% *ee*) and the catalyst could be recycled up to three times without any major loss of selectivity or yield, although the reaction times needed to attain the same conversion (95%) were markedly longer for the third run. Treating the catalyst with pyridine in between cycles in order to stabilize the Ru(II)-salen bis-pyridine complex was found to minimize catalyst deactivation and shorten the reaction times for the following runs. 

### 4.5. Ring Opening of Epoxides

**Scheme 10:**
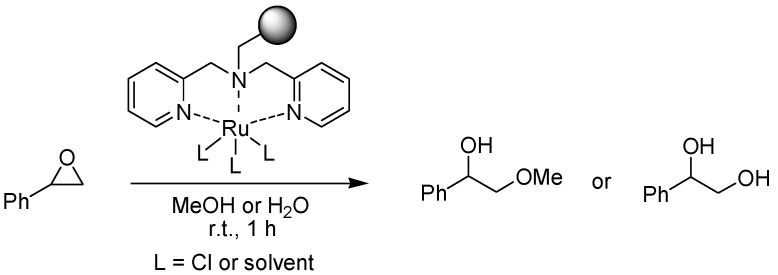
Ruthenium-catalyzed ring opening of epoxides with methanol or water, exemplified with styrene oxide as the substrate.

Ring opening of epoxides using a supported cobalt catalyst has been described earlier in this review (see Section 2.1), but the same reaction can also be carried out with ruthenium catalysis, as demonstrated by Kim and Lee [30]. A ruthenium(III) complex was anchored to a polymer-bound bis(2-picolyl)amine ligand and applied in the reaction of both aromatic and aliphatic epoxides with either methanol or water with essentially complete conversion (Scheme 10). Styrene gave a rapid reaction (1 h), while other epoxides required somewhat longer reaction times (2.5–35 h) and 1,2-epoxy-hexane needed 200 h for complete conversion. The polymer-bound catalyst could be recycled up to ten times with no loss of catalytic activity. The same types of reactions could also be carried out with a supported iron catalyst, although some leaching was observed in the case of hydrolysis.

## 5. Rhodium

### 5.1. Conjugate Addition

**Scheme 11:**
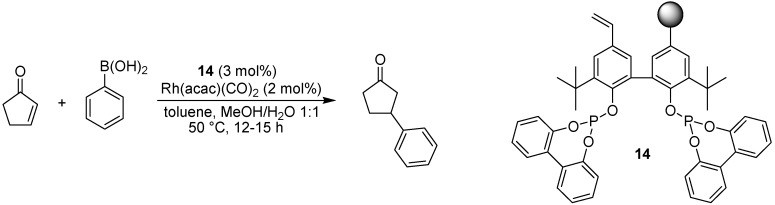
Rhodium-catalyzed conjugate addition of phenylboronic acid using a supported catalyst.

As mentioned in the introduction, catalysts attached to soluble polymeric supports are not covered in this review, but one exception will be made here. Jana and Tunge have developed a polymer-supported diphosphite ligand named JanaPhos (**14**, Scheme 11), and applied this in the rhodium-catalyzed conjugate addition of boronic acids to enones [31]. TEMPO-mediated living free radical polymerization allowed careful control of the molecular weight of the polymer, affording a support that was soluble in solvents such as toluene and dichloromethane but not in hexane or methanol. Recovery of the polymer was thus effected by precipitation with methanol at the end of the reaction. Water was found to be important in the reaction; without this protic cosolvent the yields dropped markedly. A wide range of of both enone coupling partners as well as boronic acids were investigated, affording the desired conjugate addition product in high yields (75-92%). An advantage of this method is that only 1.3 equivalents of boronic acid compared to the enone are needed, while corresponding reactions using heterogeneous catalysts require 4-5 equivalents.

### 5.2. Rhodium-Catalyzed Carbonylation, Hydroformylation and Hydrogenation

**Scheme 12:**
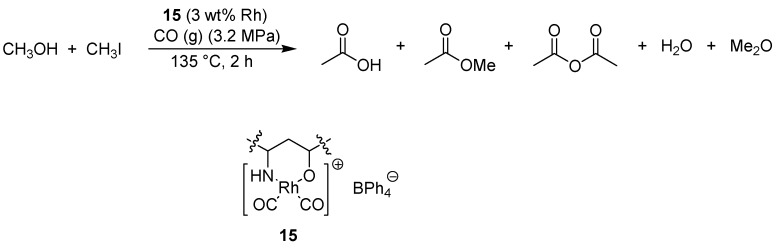
Carbonylation of methanol to form acetic acid and other products.

Yuan and co-workers used a polymer-supported rhodium complex for the carbonylation of methanol to prepare acetic acid [32]. The solid support was prepared from 2-vinylpyridine and ethylene glycol acrylate via crosslinking copolymerization, and subsequently treated with [Rh_2_(CO)_4_]Cl_2_ followed by tetraphenylboron to form the chelating complex **15** (Scheme 12). Acetic acid could then be formed from methanol under a carbon monoxide pressure of 3.2 MPa at 135 ºC. 

**Scheme 13:**
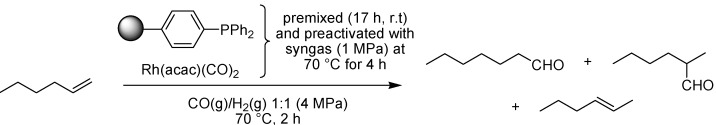
Hydroformylation using a polymer-bound rhodium catalyst.

Fujita *et al*. have prepared a catalyst from Rh(acac)(CO)_2_ and polymer supported triphenylphosphine, and applied it in the hydroformylation of 1-hexene with syngas (Scheme 13) [33]. The reaction was carried out both in supercritical carbon dioxide and in organic solvents such as toluene and ethyl acetate, where scCO_2_ was found to be superior in terms of reactivity and selectivity of the hydroformylation. The CO_2_-pressure was also found to affect the reaction, with a higher pressure affording more 2-hexene at the expense of the branched hydroformylation product. Blechert and Buchmeiser have also applied a polymer-supported rhodium catalyst in hydroformylation [34]. The support used was an amphiphilic resin, prepared via ring opening metathesis polymerization, with a pendant dipyridyl-2-ylamide moiety functioning as the ligand to rhodium. Hydroformylation of 1-octene was performed under micellar conditions with good results, the supported catalysts affording a higher selectivity for the linear product than the homogeneous analogue.

**Scheme 14:**
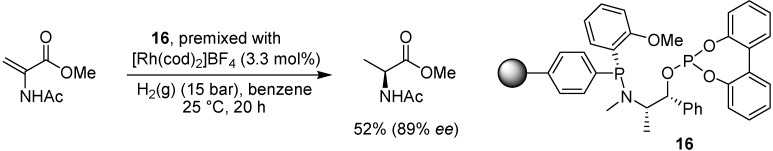
Polymer-supported chiral bidentate phosphorus ligands in the asymmetric hydrogenation of methyl α-acetamidoacrylate.

Reduction reactions are not treated to any great extent in this review, but one example of a successful asymmetric hydrogenation reaction will be included here. Kamer and co-workers have evaluated polystyrene-supported bidentate aminophosphane-phosphite and -phosphinite ligands in the asymmetric hydrogenation of conjugated methyl esters [35]. A library of resin-bound ligands was prepared in borane protected form and deprotected with diethylamine prior to treatment with a solution of [Rh(cod)_2_]BF_4_ to form the hydrogenation catalyst. Three different methyl esters were used as substrates in the screening of the ligands and the outcome both in terms of yield and stereoselectivity varied strongly depending on the structure of the ligand. One of the more successful examples, using polymer-bound ligand **16**, is shown in Scheme 14.

## 6. Palladium

Palladium is probably the most versatile of elements for application in organic synthesis, and it is therefore not surprising that the majority of reports concerning supported organometallic reagents for synthetic purposes deal with complexes involving this metal. For earlier reports on polymer supported palladium complexes, we refer the readers to a comprehensive review by Bräse [6]. Methods for carbon-carbon cross-coupling such as the Suzuki, Heck and Sonogashira reactions are the most common applications for polymer bound palladium complexes. In some cases the catalyst described has been developed for only one of these reactions, in some cases for two or all three; the text below has been sub-divided accordingly. Other important methods in this area are the palladium-catalyzed allylic substitution reaction, generally carried out in an asymmetric sense, as well as C-N bond formation methodology. For the latter reaction, reports using solid supported catalysts are scarce at the moment, but this is likely to change in the near future considering the utility of this reaction.

### 6.1. Allylic Substitution

**Scheme 15:**
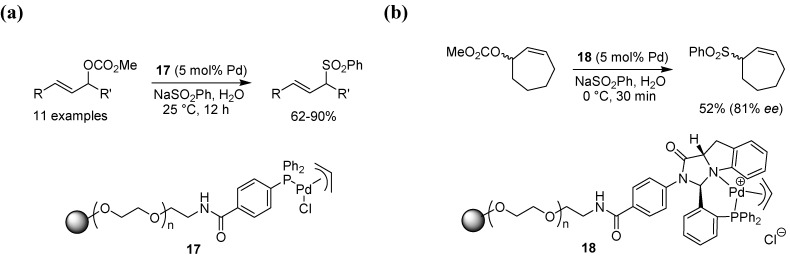
**(a)** Allylic sulfonylation using a Tentagel-supported palladium catalyst. **(b)** Asymmetric allylic sulfonylation of a cycloheptenyl carbonate substrate.

Several methods for the allylic substitution using polymer-bound catalysts exist, in general involving the use of a chiral or non-chiral ligand anchored to a solid support that is subsequently treated with a palladium reagent before application in catalysis. These methods will be briefly summarized here. Uozumi and Suzuka performed allylic sulfonation under aqueous conditions using two different Tentagel-supported phosphine palladium catalysts [36]. π-Allyl complex **17** (Scheme 15 a), anchored to the PS-PEG resin via an aromatic amide, was efficient in the sulfonylation of both acyclic as well as cyclic carbonates, and could also be recycled several times without any loss of activity. The chiral complex **18** (Scheme 15b) was applied towards the asymmetric allylic sulfonylation of cycloheptenyl carbonate with up to 81% ee, while a smaller ring size in the substrate, *i.e.* cyclopentenyl or cyclohexenyl gave product with lower enantioselectivity (33-45% ee). The same catalyst was also applied in the desymmetrization of various *meso* compounds via allylic substitution, affording up to 99% ee when phenol was used as the nucleophile [37].

**Scheme 16:**
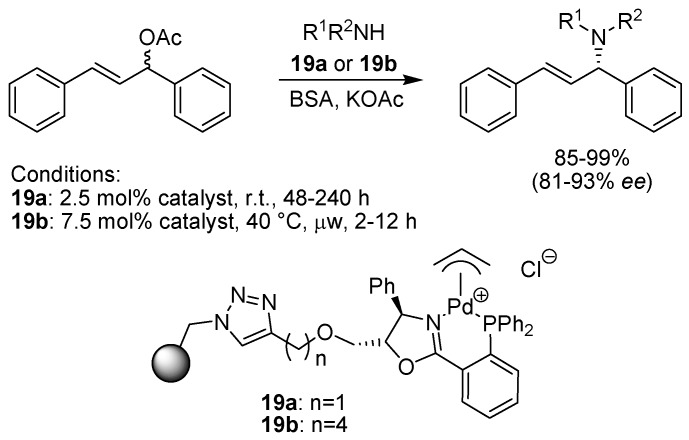
Palladium-catalyzed allylic amination using a supported PHOX-ligand.

Kamer and co-workers investigated the use of polymer-bound phosphoramidites, phosphites and related ligands in the asymmetric substitution of an allylic acetate using dimethylmalonate as the nucleophile, but found that although the conversion was high, the enantioselectivity was rather modest and not at the same level as seen for the solution phase counterparts [38]. Better results were obtained by Vidal-Ferran, Pericàs and colleagues who applied polymer bound diphenylphosphinooxazoline (PHOX) ligands in enantioselective amination of allylic acetates [39]. The chiral ligand was attached to the polystyrene support using a click reaction (Scheme 16). Reference ligands containing the same spacers and triazole connecting unit were also prepared for comparative solution phase studies. Several different parameters, such as the spacer length (**19a** and **19b**), presence or absence of potassium acetate, and the nature of the counterion were studied. Reactions involving the polymer-bound complexes in general gave both high yields and good to excellent enantioselectivities with a range of amine nucleophiles. A longer spacer length (as in **19b**) was found to give products in somewhat higher enantiomeric excess, and the use of microwave heating allowed the reaction times to be shortened substantially. The optimal ligand was also tested in a continuous flow system, adapted for use in a microwave reactor. Although the conversion dropped somewhat, the enantioselectivity still remained on par with earlier results.

### 6.2. Suzuki Coupling

**Scheme 17:**
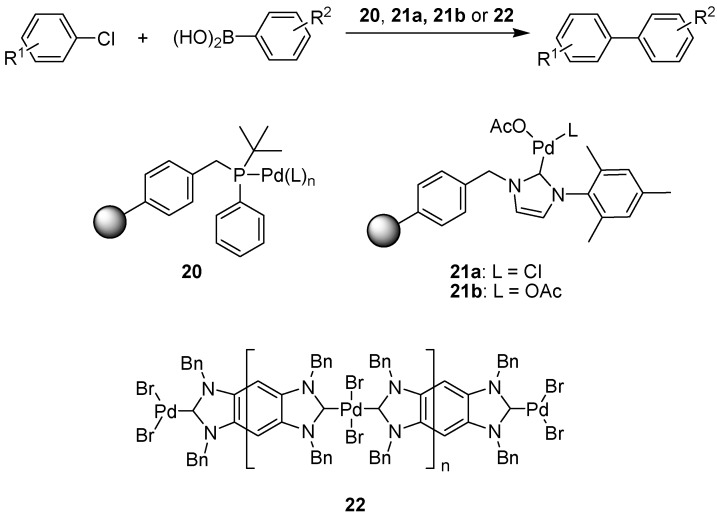
Polymer-supported palladium catalysts developed for the Suzuki coupling of aryl chlorides with boronic acids.

There is a plethora of methods for performing the Suzuki reaction on solid phase, either using polymer bound catalysts or with the substrate tethered to a support, thus only a few more recent methods will be mentioned here. Several groups have targeted the problem of coupling aryl chlorides with boronic esters, the chlorides in general being more problematic than the corresponding aryl iodides or bromides, but of interest as coupling partners due to their lower price and easier availability. Scheme 17 shows some of the more recent polymer supported catalysts developed for this purpose. 

Air-stable palladium complex **20** (Scheme 17) was prepared by Becht and co-workers and applied in the formation of biarylic compounds using as little as 0.4 mol% palladium in the reaction [40,41]. Reaction conditions involved the use of cesium fluoride as the base and reflux in toluene containing a small amount of water for 20 hours. The catalyst could be recycled up to seven times without any loss of efficiency. Lee and colleagues instead applied a carbene bound to macroporous polystyrene as the ligand to palladium in similar coupling reactions, albeit with somewhat different reaction conditions [42]. When complex **21a** and phenylboronic acid were heated together with either *ortho*- or *para*-substituted aryl chlorides in a 2:1 mixture of dimethylformamide and water, using potassium carbonate as the base, the desired coupling products were produced in reasonable yields. The methodology was also applicable to aryl iodides and aryl bromides. 

A related polymer bound palladium species was reported by Zeng *et al*., who prepared a soluble silver carbene polymer that could be used as a carbene transfer reagent for the preparation of catalyst **21b**, also for application in the Suzuki coupling of aryl chlorides [43]. By using isopropanol as the solvent, the polymeric support was rendered non-soluble. Performing the reaction at 80 ºC with sodium *tert*-butoxide as the base afforded biaryl products in high yields after 8-18 h. A slightly different approach was taken by Karimi and Akhavan, who prepared a main-chain NHC-palladium polymer (**22**) where the palladium catalyst is incorporated into the polymer backbone itself [44]. The Suzuki reaction of both activated and deactivated aryl chlorides could be performed in water at 80-90 ºC with as little as 0.05 mol% of catalyst **22**, albeit with rather long reaction times. An aryl fluoride was also coupled successfully using this method.

**Figure 1:**
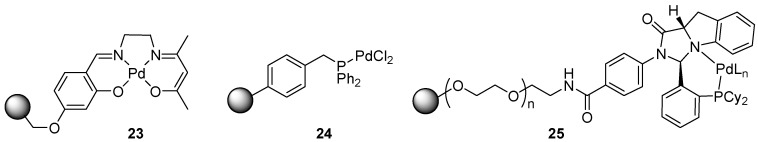
Supported catalysts developed for Suzuki reactions in water.

Continuing on the topic of Suzuki reactions in water using supported catalysts, Phan and Styring used a salen-palladium complex attached to Merrifield resin (**23**, Figure 1) for the Suzuki reaction of aryl bromides with phenylboronic acid under aqueous conditions [45]. The reaction conditions were optimized carefully and a 30:1 mixture of water and toluene was found to be the optimal solvent, with a reaction time of 24 h and heating to 90 ºC affording coupling products in high yields. Notable is that no phosphine ligand is required for this reaction and very little leaching of palladium takes place (less than 1 ppb). Bai and Wang applied palladium chloride attached to polymer-supported diphenyl-phosphine **24** in the coupling of sodium tetraphenylborate with various aryl bromides using water as the solvent in conjunction with focused microwave irradiation [46]. Potassium carbonate was found to be the optimum base, and surprisingly enough, the use of only water as the solvent gave better results than various mixtures of water and organic solvents (benzene, toluene, ethanol). The catalyst loading in the reaction corresponded to 1 mol% palladium, and the catalyst could be recycled up to ten times without any deterioration of its performance. Both electron rich and electron poor aryl bromides were investigated and a few different heterocyclic bromides were also included in the study, with yields in the range of 87-95%. The same catalyst was also used for Suzuki-like coupling of aryl halides with triarylbismuth reagents, albeit not in water in this case [47]. Asymmetric Suzuki couplings under aqueous conditions have also been reported by Uozumi et al. using a chiral aminophosphine ligand attached to a PS-PEG support (**25**) [48]. *Ortho*-substituted naphthyl chlorides and bromides were reacted with *ortho*-substituted naphthylboronic acids to form axially chiral binaphthyl products in good yields and high enantioselectivities (88-94% *ee*). This is quite a remarkable achievement considering that the reaction was carried out using only water as the solvent.

### 6.3. Sonogashira Coupling

**Scheme 18:**
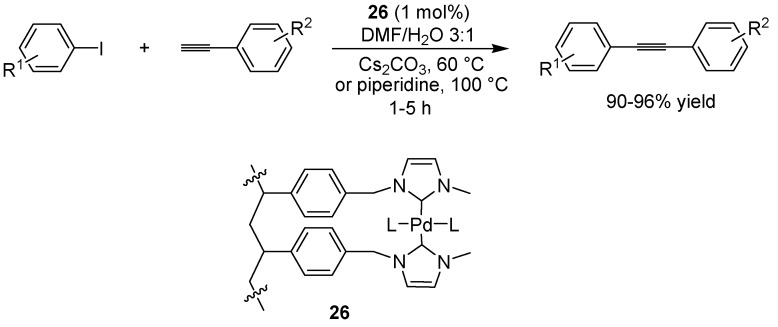
A polymer-supported *N*-heterocyclic carbenes as ligand to palladium in the copper-free Sonogashira coupling of aryl iodides with aryl acetylenes.

As for the Suzuki reaction, many methods already exist for performing the Sonogashira reaction using supported palladium catalysts. Much of the recent focus lies instead in adapting the copper-free version of this reaction to solid phase conditions. Copper can cause side reactions such as oxidative dimerization of the alkyne [49] and it is thus of interest to exclude the commonly used copper(I) iodide from the reaction protocol if possible. Lee and co-workers investigated the use of bidentate NHC-palladium complexes (**26**, Scheme 18), attached to a core-shell polymeric support, in the copper-free Sonogashira reaction [50]. For electron-deficient aryl iodides, the use of cesium carbonate as the base at 60 ºC was found to be the optimal, while for electron-rich aryl iodides, the conditions were changed somewhat, using piperidine at 100 ºC instead. Yields were in all cases in the range of 90-95%. Two aryl bromides were also included in the study, as well as one aliphatic alkyne. These also afforded the desired product, although the aliphatic substrate required a longer reaction time and gave the product in a slightly lower yield (80%). Luo and colleagues have reported a similar supported palladium-NHC complex for the Suzuki coupling of arenediazonium tetrafluoroborate salts with arylboronic acids, where the coupling reaction could be carried out at room temperature, using ethanol as the solvent [51].

Uozumi and colleagues applied the PS-PEG-tethered catalyst **17** [Scheme 15(a)], also applied for allylic sulfonylation, in the copper-free Sonogashira reaction under aqueous conditions [52]. The optimal base for most reactions was found to be triethylamine, although carboxy-substituted aliphatic terminal acetylenes fared better when cesium hydroxide was used instead. Aryl iodides as substrates in conjunction with phenyl acetylene as the alkyne in general gave high yields of coupling products under mild reaction conditions (40 ºC), while aryl bromides required a higher reaction temperature to afford product. Two aryl chlorides were also tested and gave product but in rather poor yields. The coupling of aliphatic carboxy-substituted alkynes was feasible, albeit in moderate yields.

**Scheme 19:**
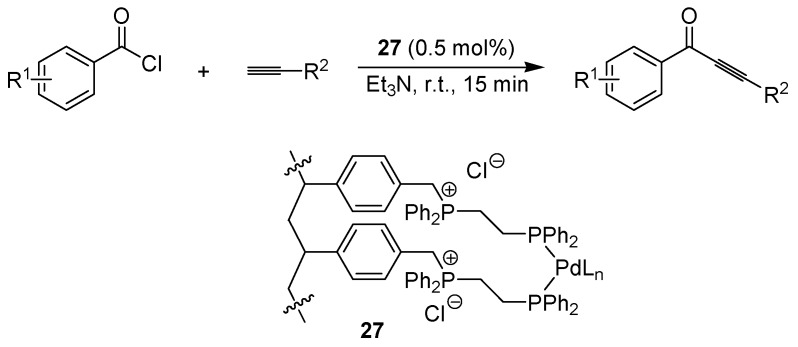
Palladium-catalyzed coupling of aroyl chlorides with terminal alkynes.

Bakherad *et al*. prepared a palladium(0) diphenylphosphinoethane complex attached to a poly-styrene support (**27**, Scheme 19) and applied this in the copper-free Sonogashira coupling with good results [53]. Various solvents and bases were screened, and running the reaction in neat piperidine at room temperature with 1 mol% **27 **was found to be the best option. Both aromatic and aliphatic alkynes were investigated, while the aryl halide was limited to aromatic iodides. The same catalyst was subsequently used for coupling terminal alkynes with aromatic acid chlorides, using triethylamine as the combined base/solvent (Scheme 19) [54]. The possibility for recycling the catalyst were also studied and found to be good, with only a small drop in product yield after ten reaction cycles. Both types of coupling reactions were run under aerobic conditions.

### 6.4. The Heck Reaction

**Scheme 20:**
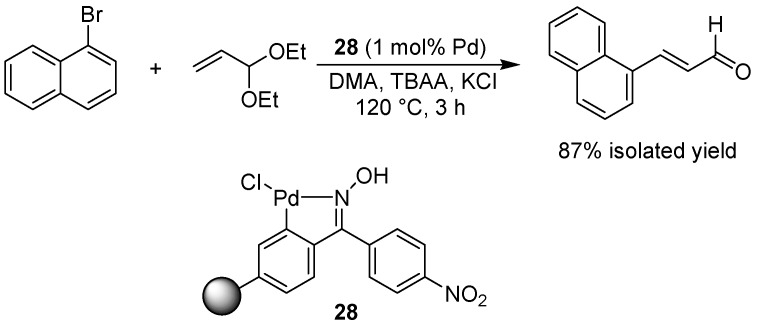
Heck coupling of acrolein with 1-bromonaphthalene using a supported Kaiser oxime palladacycle.

The palladium-catalyzed coupling of acrolein with aryl halides using the Heck reaction is usually fraught with difficulties due to the propensity for acrolein to polymerize during the reaction conditions. By using acrolein diethyl acetal as the substrate under so called Cacchi conditions [55], Alacid and Nájera have shown that cinnamic aldehyde derivatives can be prepared from aryl halides via the Heck reaction using a polystyrene-supported Kaiser oxime palladacycle (**28**, Scheme 20) [56]. Aryl iodides, bromides and chlorides could all be used as the aromatic halide precursor. 3-Arylpropanoates could also be prepared under somewhat different reaction conditions, using the same catalyst. The efficiency of the polymer-supported catalyst was found to be in line with the corresponding unsupported catalyst, and leaching of palladium into solution was negligible (0.06-0.08 ppm). A more complete study from the same group includes the use of other alkene substrates for the Heck reaction [57]. Other examples of Heck reactions using polymer-bound catalysts have been reported by Beletskaya *et al.* [58], involving the use of poly(*N*-vinylimidazole) as the polymer, and Wu and co-workers [59], who used mesostructured polymeric supports linked to a palladium catalyst.

**Scheme 21:**
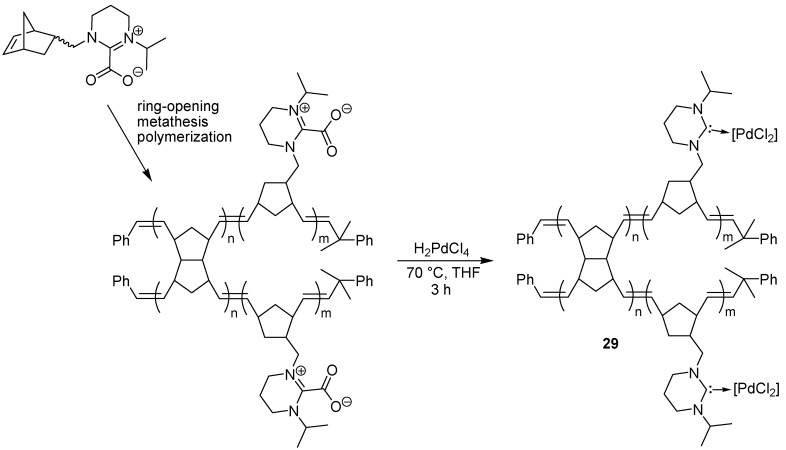
Preparation of a polymer-bound CO_2_-protected *N*-heterocyclic carbene and its direct conversion into a supported palladium catalyst.

**Scheme 22:**
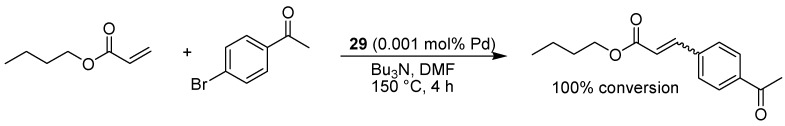
Application of polymer-supported catalyst **29 **in the Heck coupling of butyl acrylate with 4-bromoacetophenone.

*N*-Heterocyclic carbenes (NHCs) are useful ligands to metals like palladium, but the free carbenes are unstable entities that can be difficult to handle. Carbon dioxide can be used as a protecting group for carbenes, however [60,61]. This practical technique has been exploited in a solid phase context by Pawar and Buchmeiser, who prepared a carbon dioxide adduct of a tetrahydropyrimidine-derived carbene attached to a polymeric support, which in turn was prepared *in situ* via ring-opening metathesis of a pendant norbornene functionality attached to the protected carbene [62]. The protected polymer-bound ligand was then converted to a supported metal complex, the metal being rhodium, iridium or palladium. The palladium-supported complex **29** (Scheme 21) was found to be very efficient as a catalyst in the Heck reaction of styrene and butyl acrylate with various aryl bromides, with turn-over numbers as high as 100,000 (TOF 25,000) (Scheme 22). The corresponding iridium complex was applied in a hydrogen transfer reaction, while the rhodium complex was used for the polymerization of phenyl acetylene.

### 6.5. Catalysts Applicable Towards Several Cross-Coupling Reaction Types

**Figure 2:**
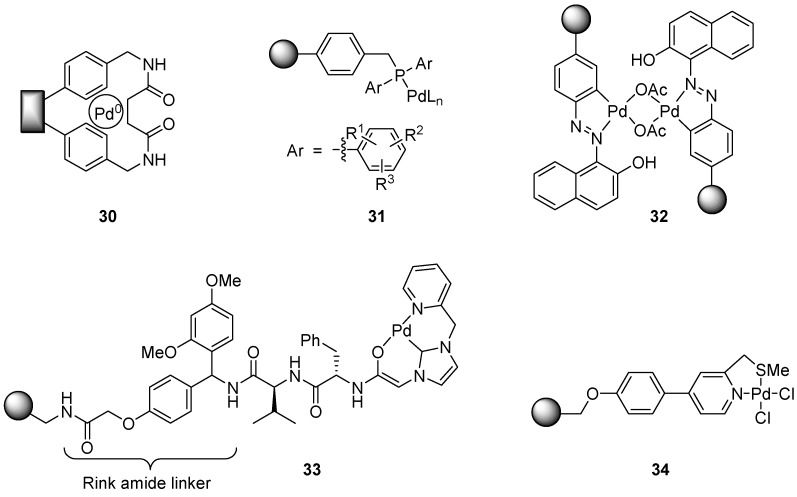
Catalysts developed for several types of cross-coupling reactions.

Several catalysts have been developed for more than one cross-coupling reaction; a brief overview of these will be given here. Bradley and co-workers have developed plugs where functionalized resin is embedded within high-density polyethylene, to afford polymer-bound reagents in a format more easy to handle than the classical polymer beads [63]. In a more recent report, aminomethylstyrene resin plugs were treated with palladium(II) acetate; the loaded Pd(II) was subsequently reduced to Pd(0) using hydrazine and finally entrapped into the resin via cross-linking with succinyl chloride, to form a catalyst with the structure shown in Figure 2 (structure **30**) [64]. Analysis of the plugs with a microscope revealed that smaller palladium nanoparticles were found at the edge of the beads with the bead size increasing towards the middle of the bead. The plugs were subsequently evaluated in three different cross-coupling reactions, i.e. the Suzuki, Heck and Sonogashira reactions, mainly involving aryl iodides, with good results. Attempted coupling of an aryl bromide in the Heck reaction was less successful. The plugs could be recycled up to three times without any major loss of activity. Schweizer *et al*. applied polystyrene-supported diarylphosphine palladium complexes **31** in the same three cross-coupling reactions, also with success [65]. Best results were obtained when the aromatic substituent was *ortho*-tolyl (Sonogashira, Suzuki) or *meta*-tolyl (Heck). The recyclability of the catalysts was found to be excellent and the activity was similar to that of palladium(0) tetrakistriphenylphosphine in solution.

Islam *et al.* have performed Suzuki couplings as well as copper-free Sonogashira reactions in water using a palladium(II) azo complex tethered to polystyrene (Figure 2, structure **32**) [66]. The use of potassium carbonate as the base was found to be important for the yield, and after optimization both aryl bromides and aryl iodides could be applied in the two reactions with good results. Aryl chlorides only gave coupling product in the Suzuki reaction, however. Worm-Leonhard and Meldal have prepared a rather unusual class of supported palladium catalysts, where a ligand system incorporating a pyridine ring as well as one or two *N*-heterocyclic carbene moieties are attached to a PEGA-resin (a polyethylene glycol dimethylacrylamide copolymer) using a dipeptide tether and a Rink amide linker (monocarbene ligand shown in Figure 2, see structure **33**) [67]. The catalytic system **33** was stable enough to allow Suzuki-coupling to be carried out in water at 50-65 ºC for 6 h. Only aryl iodides and bromides were used as substrates. The corresponding dicarbene catalyst was cleaved off the resin before application, and employed in microwave-mediated Suzuki coupling, allowing the reaction time to be reduced to 10 min, albeit at a higher reaction temperature (90-100 ºC). Catalyst **33** was also used in the coupling of aryl iodides with terminal alkynes, i.e. the Sonogashira reaction, with good results. The chirality of the tether was not exploited in this work, but opens up the possibility of investigating this class of ligands in asymmetric metal-catalyzed transformations. Jones, Canty and co-workers have exploited palladium complex **34**, with an *N*,*S*-donor ligand, in Suzuki and Heck reactions, comparing different types of support materials [68]. Aryl bromides and aryl iodides functioned well in both reactions, irrespective of the support used, *i.e*. Merrifield resin, Wang resin or a ground macroporous polymer monolith material, with the Wang resin providing the highest turnover numbers (up to 19950). The use of aryl chlorides afforded very little product, however. The reactions could also be carried out in capillary microreactors filled with monolith, with good results, albeit somewhat lower yields when using an aryl bromide as the substrate. Basu, Almqvist and co-workers have attached palladium to a polyionic Amberlite resin to provide a ligand free catalyst that can be applied in Heck, Suzuki and Sonogashira reactions [69]. Formate was used as counterion to effect in situ reduction of Pd(II) to Pd(0), followed by deposition of the active palladium species on the resin surface. Heck reactions of aryl iodides with ethyl acrylate afforded the desired coupling products in up to 90% yield after 5-10 hours at 90-100 ºC. Sonogashira coupling of aryl iodides was also effected in high yields, up to 96% when using a 3-methyl substituted aryl iodide. Suzuki coupling of aryl bromides using this type of catalyst gave yields in the range of 80-90%, and diarylation of 1,2-bromobenzene could also be effected in 55% yield. Investigation of the recyclability of the catalyst in the Suzuki reaction showed that diarylation of 1,4-dibromobenzene could be performed up to five times without any great loss of activity. The palladium content was followed by x-ray photoelectron spectroscopy (XPS), measuring the Pd/N ratio, and showed that although some leaching occurred in the first cycle, the level then remained essentially the same throughout the following cycles.

A brief mention will also be made of some additional methods, without going into much detail. Dahan and Portnoy functionalized Wang polystyrene with different dendrimers, using pendant phosphines as ligands for palladium, and applied these supported catalysts in Heck and Suzuki coupling reactions [70]. A positive dendritic effect was seen in all cases, *i.e.* third generation dendrimer supports performed markedly better than the non-dendronized polymer or the first generation dendronized support. Finally, Zhang and co-workers investigated the use of pH-responsive core shell polymeric microspheres as supports for palladium(II) chloride [71]. The quasi-homogeneous microspheres consisted of an inner polystyrene core and an outer poly(methacrylic acid) shell, employing a pendant iminodiacetic acid unit as the ligand to palladium. Both Heck and Suzuki reactions could be carried out in water using this catalyst, recycling up to four times was possible without loss of catalytic activity.

### 6.6. Aryl Amination (C-N Coupling)

**Scheme 23:**
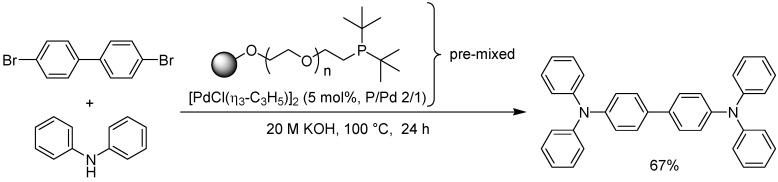
Palladium-catalyzed aryl amination under aqueous conditions.

Uozumi is one of the more established researchers in the area of catalysis using supported organometallic complexes, and in a recent paper together with Hirai [72] he describes the development of a PS-PEG supported palladium catalyst for the coupling of aryl halides with anilines using the Buchwald-Hartwig aryl amination under aqueous conditions. PS-PEG-(dicyclohexyl)phosphine as well as PS-PEG-(di*-tert*-butyl)phosphine were prepared and pre-treated with di(μ-chloro)bis(η^3^-allyl)dipalladium(II) and subjected to the catalytic amination of aryl bromides. Only the (di-*tert*-butyl) substituted phosphine palladium complex was found to be efficient in the reaction, however. Coupling of aryl bromides with diphenylamine in general afforded the desired products in high yields (85-95%) if the aryl group was *meta*- or *para*-substituted, while a substituent in the *ortho*-position in general lowered the yield. Iodobenzene as well as chlorobenzene also worked well in the reaction. The double arylation of primary anilines using an excess of aryl bromide could also be effected with excellent results. To demonstrate the utility of the method, several TPD derivatives (TPD = (*N*,*N*,*N*’,*N*’)-tetraphenyl-1,1’-biphenyl-4,4’-diamine), of interest as organic materials in optoelectronic devices, were prepared (one example is shown in Scheme 23). Metal-contamination is generally a problem in the preparation of TPDs, but by using a polymer-bound catalyst, leaching could be kept to a minimum.

**Scheme 24:**
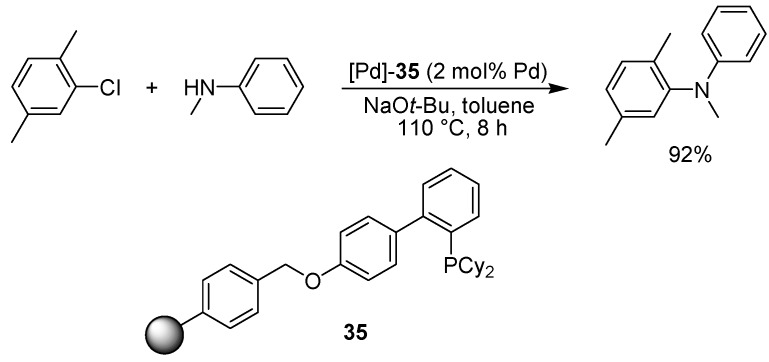
Aryl amination using a polymer-incarcerated palladium catalyst.

Kobayashi and colleagues applied a palladium catalyst, incarcerated in a polymeric structure via microencapsulation and cross-linking, in the amination of aryl chlorides (Scheme 24) [73,74]. The copolymer used for preparing the support material was equipped with pendant bulky phosphines suitable for the aryl amination reaction. A wide range of aryl chlorides could be coupled with primary, secondary as well as anilinic amines in good yields and with a low degree of palladium leaching. No external phosphine was needed for the reaction to proceed. A brief mention will also be made of a study by Kiil and co-workers, who evaluated four different commercially available so called FibreCat catalysts, consisting of a polypropylene backbone with appended phosphine ligands coordinated to palladium [75]. The authors found that reused supported catalyst afforded more side products in ensuing reactions, most likely due to leaching of palladium and phosphine from the FibreCat system. Highest selectivity for the desired aryl amination product was formed using a supported triphenylphosphine/palladium(II) chloride catalyst.

### 6.7. Allylic Imidate Rearrangement

**Scheme 25:**
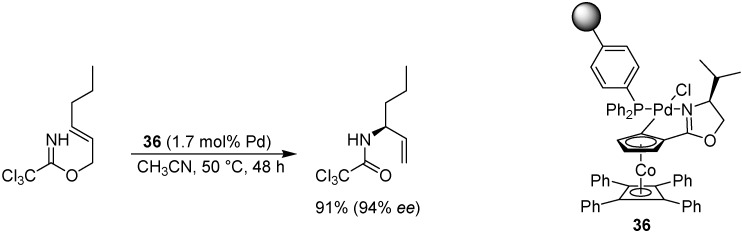
A polymer-supported palladacycle for the allylic imidate rearrangement.

Nomura and Richards have studied the asymmetric allylic rearrangement of imidates, mainly focusing on soluble catalysts, but one example of a solid supported catalyst was also included in the study [76]. Polymer-supported palladacycle **36** (Scheme 25) was developed in order to facilitate recycling of the catalyst, and was found to give approximately the same yield and selectivity as the solution phase counterpart. Sequential application was more problematic however; although the stereoselectivity remained essentially intact, the yields dropped markedly in the second and third run, most likely due to palladium deactivation.

## 7. Iridium

### 7.1. Transfer Dehydrogenation

**Scheme 26:**
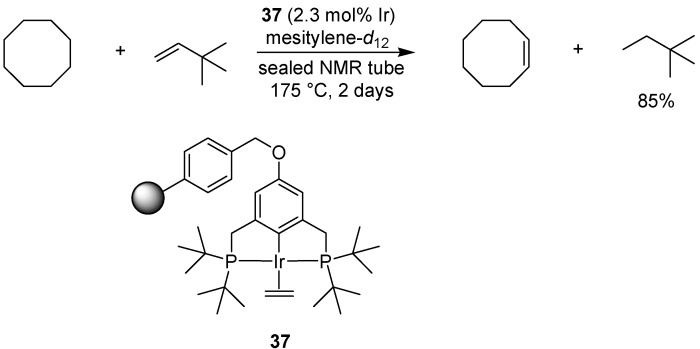
Transfer hydrogenation employing a polymer-supported iridium pincer complex.

Huang *et al.* have investigated three different types of supported iridium pincer complexes for the transfer dehydrogenation of alkanes, comparing covalent attachment of the complex to Merrifield resin or silica, to adsorption onto γ-Al_2_O_3_ (Scheme 26) [77]. Cyclooctane was used as the alkane partner in the reaction, together with *tert*-butylethylene as the hydrogen acceptor, forming cyclooctene and 2,2-dimethylbutane. Application of polystyrene-supported complex **37** in the reaction afforded 85% of 2,2-dimethylbutane after 2 days. However, a second reaction using recovered catalyst afforded only 20% product, indicating decomposition of the iridium pincer complex. Overall, the catalyst adsorbed onto γ-Al_2_O_3_ was found to be more robust and efficient than the covalently bound catalysts in this case.

## 8. Concluding Remarks

Attaching an organometallic catalyst to a solid phase has many advantages compared to running the reaction in solution, not only in terms of simplified purification but also in minimizing contamination of the final product with metallic residues, which can often be a problem and is of special importance within the pharmaceutical industry where such contaminants can affect the biological testing of potential drug candidates. Another important aspect is that of green chemistry, *i.e.* developing efficient and environmentally benign methods for organic synthesis, where the fact that many supported catalysts can be recycled a number of times without loss of efficiency constitutes an important contribution in this area. Although a number of new methods of palladium-catalyzed cross-coupling on solid phase now exist, there are many other areas in this domain that are still relatively unexplored, such as asymmetric reactions, aryl amination and reactions involving other transition metals than palladium, and we foresee a continued expansion of this inspiring field.
